# Hydraulic property variations with depth in a loess mudstone landslide

**DOI:** 10.1038/s41598-024-61890-x

**Published:** 2024-05-14

**Authors:** Wei Liu, Gaochao Lin, Qingpeng Liu, Xing Su

**Affiliations:** 1 Institute of Transportation, Inner Mongolia University, Hohhot, 010070 Inner Mongolia China; 2 School of Civil and Environmental Engineering, University of New South Wales, Sydney, NSW 2052 Australia; 3 Institute of Geological Hazards Prevention, Gansu Academy of Sciences, Lanzhou, 730000 Gansu China

**Keywords:** Loess-mudstone landslide, Hydraulic property, Soil water characteristic curve, Water retention capacity, Environmental sciences, Hydrology, Natural hazards

## Abstract

In areas where loess is distributed, landslides represent a significant geohazard with severe implications. Among these events, loess-mudstone landslides are particularly prevalent, posing substantial risks to the safety and property of local residents, and moisture plays a pivotal role as a key factor in causing these disasters. In this study, the hydraulic properties of the soils along the longitudinal section of an ongoing loess-mudstone landslide are investigated through the variation of soil water characteristic curves, which are subsequently fitted by utilizing van Genuchten model. Moreover, a comprehensive experimental investigation was conducted on the loess, mudstone, and loess-mudstone mixtures to facilitate analysis, including X-ray diffraction (XRD) analysis, scanning electron microscopy (SEM) observation, particle size distribution (PSD) analysis, along with fundamental geotechnical tests for parameter determination. It is found that mudstone and loess have distinct SWCC distribution. The SWCC of loess at various depths exhibits a similar distribution pattern due to the occurrence of landslide. The SWCC distribution of loess-mudstone mixture displays a transitional trend between the SWCC of mudstone and that of loess, and the water retention capacity increases as the mudstone content increases. The experimental findings have demonstrated notable agreement between each other and exhibited a satisfactory level of concurrence with the observed phenomena in geological surveys.

## Introduction

Loess, a distinctive Quaternary geological formation, is found abundantly worldwide, spanning across continents such as Asia, Europe, and North and South America^1–3^. Notably, China alone holds approximately 631,000 km^2^ of loess, encompassing roughly 6.6% of the country's total land area. The most significant accumulation of which, about 317,000 km^2^, shapes the renowned Loess Plateau, making it the largest such formation on the planet^4–6^. Loess has typical collapsibility, structuredness and water sensitivity. Loess exhibits favorable mechanical attributes, yet its structural integrity deteriorates significantly upon contact with water, resulting in rapid strength disaggregation and subsequent soil instability. The convergence of several contributing factors, namely the seasonal precipitation concentration stemming from the monsoon climate, augmented irrigation demands associated with urbanization, and underlying regional seismic activity driven by the Himalayan–Tibetan Plateau's tectonic movement, have collectively precipitated a heightened frequency of geological catastrophes within the Loess Plateau, seriously threatening the lives and property of local people. The most common type of landslide disaster in the Loess Plateau area is observed along the interface between loess and underlying bedrock, primarily attributable to that the bedrock of loess frequently consists of impermeable argillite or mudstones^4,7,8^. Consequently, the impediment of groundwater flow leads to the accumulation of a saturation zone at the interface between the loess and bedrock and triggers the subsequent landslides. Given the extensive occurrence of this type of landslide, many scholars consider it as a distinct and significant classification when categorizing loess landslides, exemplified by Li et al.^9^, Wu et al.^10^, Xu et al.^11^, etc. For the sake of brevity, it is referred as 'loess-mudstone landslide' in the subsequent description. Famous historical instances include the Saleshan Landslide that transpired on March 7, 1983, resulting in a total sliding volume of 3.10 × 10^7^ m^3^ and complete devastation of three neighboring villages, tragically causing 237 fatalities^12^. Another noteworthy example is the Huangci Landslide on January 30, 1995, with a sliding volume reaching 6 × 10^6^ m^3^. Fortunately, due to stringent monitoring and accurate landslide prediction, this event resulted in no casualties^13^. It is evident that gaining a comprehensive understanding of landslide disasters and thereby proposing effective prevention and forecasting measures holds immense importance in safeguarding the lives and properties of local people.

The existing research on loess-mudstone landslides primarily concentrates on investigating the distinct behaviors of loess and mudstone when subjected to dynamic loading from various angles. For example, Zhang et al.^14^ conducted centrifuge shaking table tests and numerical simulations to investigate the dynamic behavior of the loess-mudstone slope under earthquakes, and they revealed the effects of height, lithologic and surface on PGA amplification factors and determined the corresponding displacement of loess and mudstone. Chen et al.^15^ applied large-scale shaking table test to investigate the failure mechanism of loess-mudstone landslides and they pointed out that the energy response was completely different between the two media. Similar attempts can also be found in the work of Huang et al.^16^, Jia et al.^17^, Lin and Wang^18^, Liu et al.^19^, Srilatha et al.^20^, Wang and Lin^21^, and Yu et al.^22^, to name a few. Moreover, the existing literature also focus on examining the variations in the mechanical properties of loess and mudstone under diverse circumstances. Wen et al.^8^ studied the residual shear strength of weathered red mudstone subjected to lixiviation by irrigation water and attributed the strength reduction to the particle disaggregation and the leached soluble salts. Zhang et al.^23^ investigated the effects of rainwater softening on the mudstone via laboratory tests, and they argued that shear strength of mudstone experienced reduction under the condition of rainwater infiltrates, which was attributed to structure variation of clay minerals. Wang et al.^24^ carried out ring shear tests and numerical simulation to study the trigger mechanism of loess-mudstone landslides, and they indicated that peak strength and residual strength decreased with increasing moisture content.

While the importance of water in loess mudstone landslide is widely acknowledged, the existing literature contains a modest amount of research on hydraulic properties, and even fewer studies on water retention properties. Loess-mudstone landslides exhibit characteristics such as large sliding scale, slow sliding speeds, unstable sliding bodies, and pronounced recurrence patterns, which contribute to their prolonged development process. Throughout this process, the hydraulic conditions within the soil undergo gradual transformations, rather than remaining consistently saturated. The alterations in water content profoundly impact the properties of loess, thereby influencing the evolution of loess-mudstone landslides. Hence, comprehending the hydraulic properties of the soil holds substantial theoretical and practical importance. The Soil Water Characteristic Curve (SWCC), also known as Soil Water Retention Curve (SWRC), offers crucial relevant information as it represents the soil's ability to hold water across a range of moisture conditions, from unsaturated to saturated states. Specifically, the curve is presented as a plot of the logarithm of either matric suction or total suction against either the degree of saturation, the gravimetric water content, or the volumetric water content. Moreover, SWCC serves as a fundamental element for accurately characterizing the properties of unsaturated soil, including strength, permeability, and volume change. These aspects significantly influence the stability of landslides. Therefore, gaining a thorough understanding of the SWCC in the soils associated with loess-mudstone landslides allows for a more comprehensive understanding of the characteristics inherent in this type of landslide. Additionally, it provides substantial experimental support for existing studies, facilitating further development and enhancement of relevant theoretical models. Based on this, this study focuses on an active loess mudstone landslide as the research subject. Soil samples at various depths will be collected through drilling for SWCC experiments, enabling to comprehensively investigate the longitudinal variations in soil hydraulic properties in loess mudstone landslide.

## Case study

**Figure 1:**
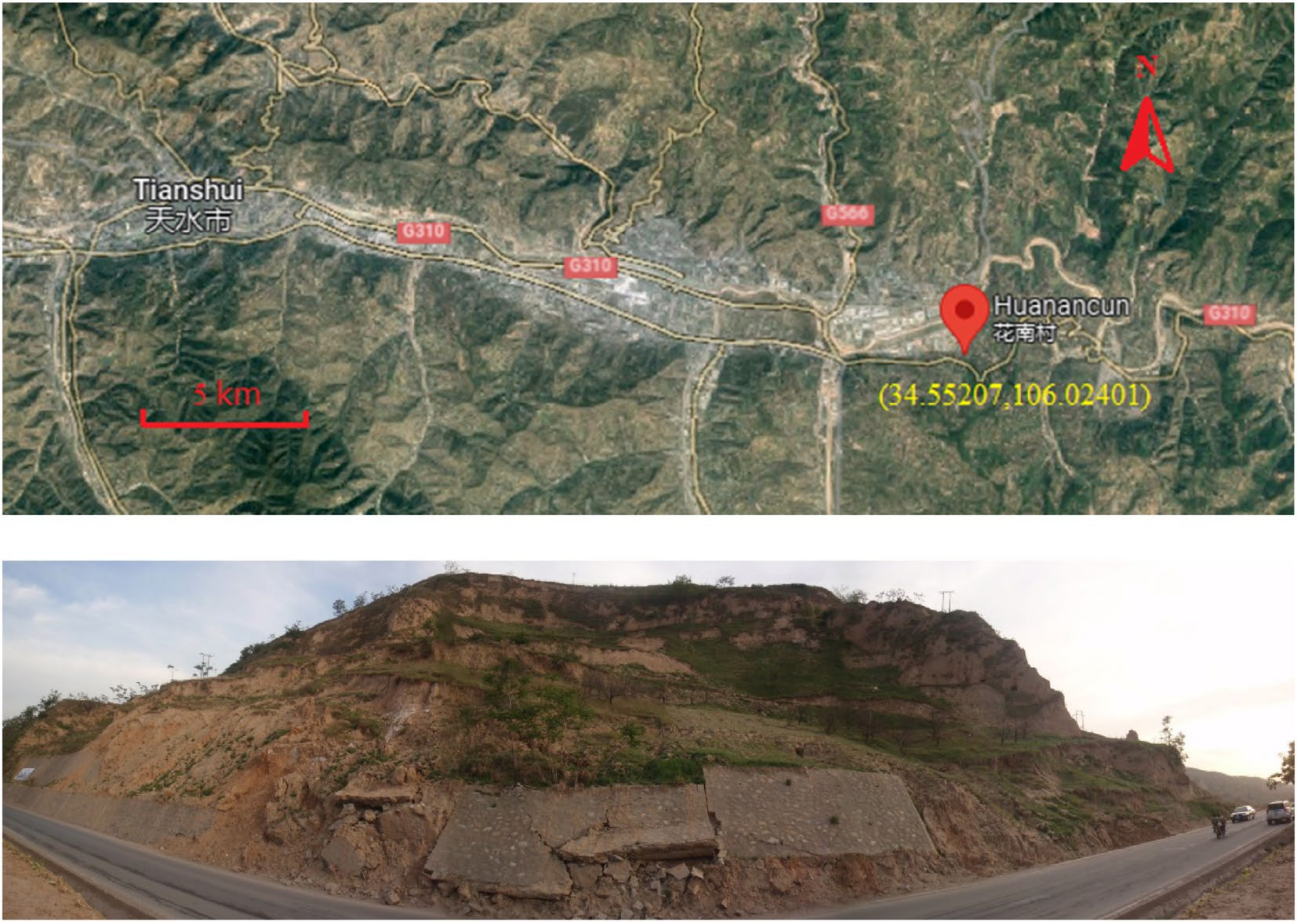
Satellite image of the Huanancun location from Google Maps^29^ and the actual picture of Huanancun landslide.

The Huanancun Landslide derived its name from its location in the Huanancun Village, Maiji District, Tianshui City. The satellite image of the Huanancun location and the actual picture of landslide are shown in Fig. 1. Geographically, it is situated in the Longzhong Basin, which is part of the Loess Plateau in western China. The dominant geological tectonics influencing this area is the Longxi Fold-Thrust Belt. This tectonic system is comprised of a series of folds, thrust faults, and shear zones that have developed since the Cretaceous period and matured during the Paleogene period, remaining tectonically active to the present day. In terms of climate, the Longzhong Basin experiences transitional conditions between a temperate semi-humid zone and a semi-arid zone due to the influence of the Liupan Mountains and the Western Qinling Mountains. Precipitation is concentrated mainly in July and August, with occasional continuous rainfall events. The annual average rainfall in the landslide area is 510.9 mm, with the maximum daily precipitation recorded at 100.3 mm. The sliding bed of the Huanancun Landslide is composed of Neogene mudstone, which has a less diverse lithology compared to Cretaceous mudstone. The layers are predominantly thick and exhibit relatively undeveloped bedding planes. Except for the edge of the basin and near the fault zone, the rock formations are mostly nearly horizontal. The mudstone is mainly overlain by Malan loess, a light grayish-yellow soil with a uniform texture and developed porosity. It possesses a loose structure and vertical joints, demonstrating significant collapsibility^4,25–28^.

**Figure 2:**
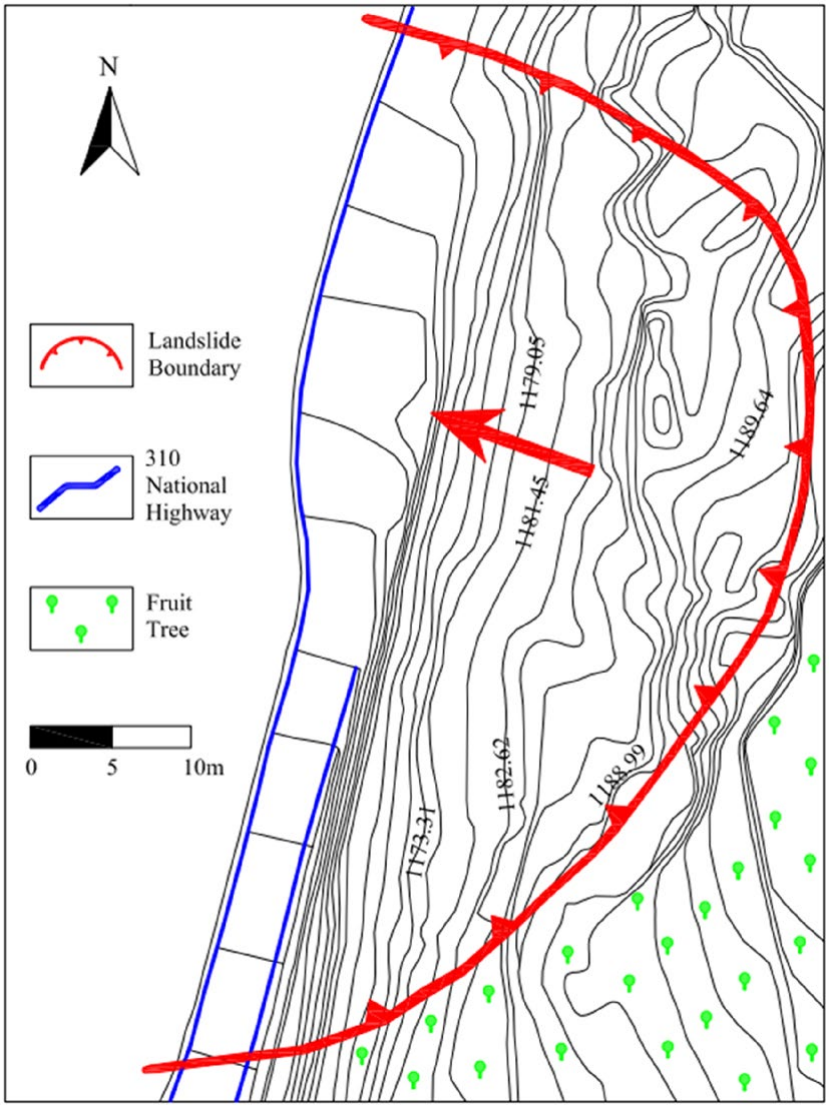
Huanancun Landslide plane mapping.

**Figure 3:**
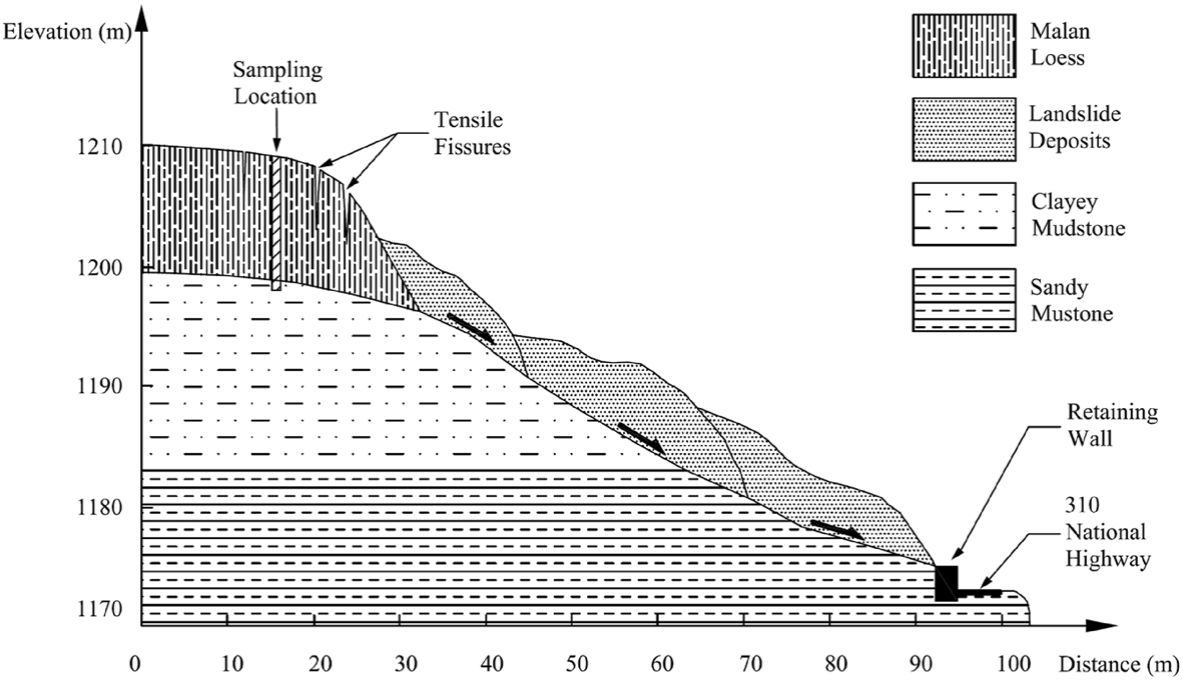
Huanancun Landslide cross sectional schematic diagram.

The Huanancun Landslide first occurred in March 2014. The landslide spans a total length of 95 m and has a width of 130 m. Its thickness ranges between 5–10 m, with an area of 1.24 × 104 m^2^ and a volume of approximately 1.24 × 10^5^ m^3^. The rear edge has an elevation of 1210 m, while the front edge stands at 1170 m, resulting in a 40 m difference in elevation. The surface of the landslide has an average slope of approximately 16°. At the trailing edge of the landslide, there are several tensile fissures perpendicular to the sliding direction. These fissures have varying widths, ranging from 0.32 m to 1.16 m, and lengths between 5.0 m to 21.5 m. These fissures cut the loess layer into uneven blocks. As a result of the landslide, all the retaining walls in the K1428 + 10 ~ K1428 + 210 section of National Highway 310 were destroyed, leaving behind accumulated landslide deposits on the road surface. The plan view and cross-sectional schematic diagrams of the landslide are shown in Figs. 2 and 3.

## Test material

The soil samples utilized in this study were taken from the loess platform that features a steep rear wall behind the primary sliding direction. To ensure the representative of soil samples, the sampling location underwent thorough inspection, and sampling points with minimal disturbance were meticulously chosen. Moreover, the sampling process was executed with utmost care to further reduce potential disturbances. The thickness of the loess layer at the sampling location is about 10 m. In order to investigate the variation in water retention capacity along the longitudinal section, soil samples are carefully collected every one meter from the ground surface. Additionally, boreholes were utilized in nearby area to obtain mudstone soil samples for comparison. To facilitate clear referencing and descriptions, each soil sample collected at specific depths was numbered according to their corresponding depths. For instance, the loess soil sample taken at a depth of 3 m was labeled as "loess 3". Since the loess layer is about 10 m thick, a total of ten groups of loess samples were collected, numbered from "loess1" to "loess10". This numbering convention will be applied in subsequent discussion of loess at different depths, as well as in the graphical presentation of the experimental results involved. Upon excavation, the soil samples were meticulously stored in sealed containers and subsequently transported to the laboratory for tests. Basic geotechnical experiments were performed on the undisturbed loess soil to ascertain the essential characteristics, which encompassed the determination of natural moisture content, density, specific gravity, plastic and liquid limits, as well as particle size distribution (PSD). For mudstone, it was thoroughly crushed by using a mortar and then conducted with the aforementioned tests.

**Figure 4:**
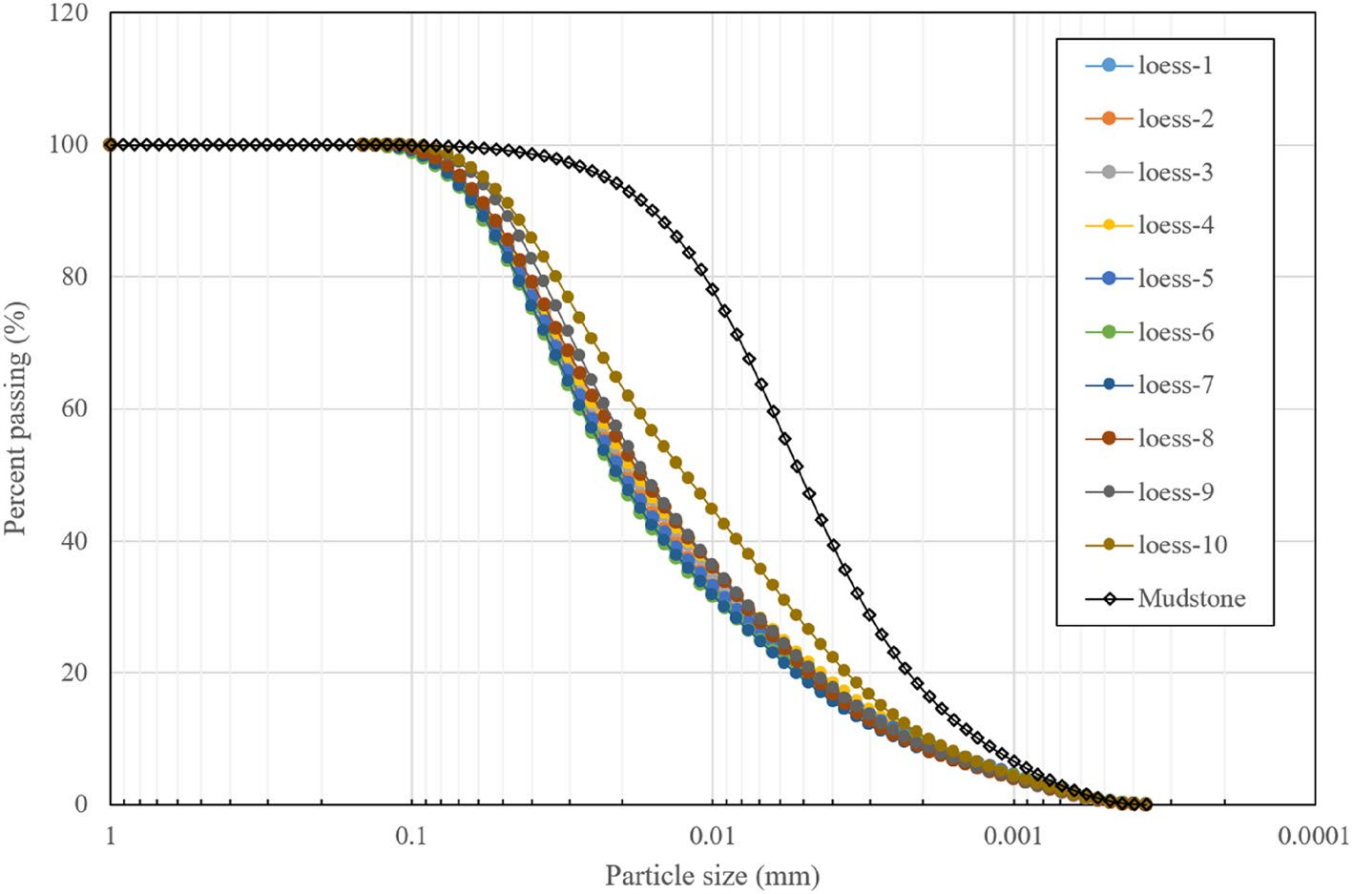
Particle size distribution curve for loess and mudstone in Huanancun, Tianshui.

The basic physical attributes of the collected soils are listed in Table 1, and the particle size distribution curves are presented in Fig. 4. The analysis of particle size results reveals that, with the exception of a slight increase in the fine-grained components of the loess sample at a depth of 10 m, the particle size curves for the remaining loess samples exhibit a consistent pattern. This consistency indicates that the particle composition within the loess layer is almost uniform. Regarding the soil sample at a depth of 10 m, it may have undergone mixing with the mudstone during the sliding process due to proximity to mudstone, thereby elevating the fine-grained soil content in this particular sample. In contrast, the mudstone samples exhibit higher contents of silt and clay, suggesting a greater presence of clay minerals within the mudstone.

**Table 1:** Basic physical indices of loess.

Soil	Specific gravity	Plastic limit (%)	Liquid limit (%)	Plastic index
Loess	2.72	19.00	29.50	10.50
Mudstone	2.74	21.23	40.68	19.45

**Figure 5:**
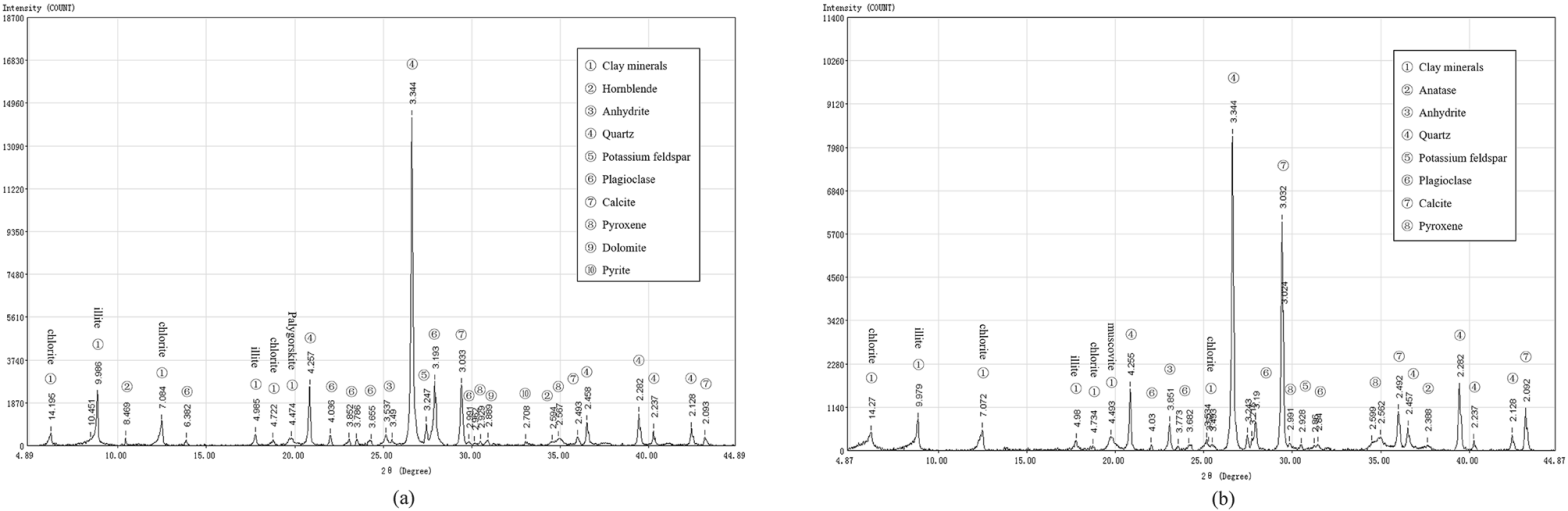
X-ray diffraction patterns of (**a**) loess and (**b**) mudstone.

In addition, mineral compositions of the loess and mudstone are analyzed through X-ray diffraction (XRD) tests, and the test results are presented in Fig. 5. The distinctive properties of clay minerals, such as low crystallinity, common isomorphic substitutions, diverse types, comparable structures, numerous mixed-layer minerals, and substantial diffraction peak overlaps, render quantitative analysis challenging. Nonetheless, qualitative assessment can be achieved through XRD experiments, complemented by particle fraction curves, to enhance comprehension of soil composition. As illustrated in Fig. 5, the non-clay mineral constituents of loess and mudstone exhibit a fundamental resemblance, primarily comprising quartz. Loess presents greater diversity in mineral types, while mudstone contains a higher proportion of calcite. Analysis of particle size distribution reveals a higher presence of clay minerals in mudstone, constituting a principal factor contributing to the marked differences in properties between the two soil types.

## Test methods

The loess samples used for SWCC experiments were obtained by carefully cutting undisturbed loess collected from site using a containing ring. However, due to the hardness, the mudstone was challenging to cut directly using a containing ring. Therefore, the mudstone was first grounded into powder before being statically compacted into the containing ring. The bulk density of the collected mudstone was determined to be approximately 2.15 g/cm^3^. Hence, for better simulation of the in-situ conditions, the remolded mudstone samples were assigned the same value.

In addition to the pure loess samples taken from various depths and the underlying pure mudstone samples, this study also included three groups of loess-mudstone mixtures. These mixtures contained varying mass percentages of mudstone, specifically 5%, 15%, and 25%. During landslides, the loess at the base rubs against and mixes with the underlying mudstone, resulting in a soil composition which is actually a combination of the two materials. The loess-mudstone landslide exhibits notable characteristics such as a prolonged activity period and strong recurrence, and the active landslide can experience renewed sliding under the influence of subsequent water immersion and/or other disturbance factors^10,11^. Therefore, the properties of the mixture at the slip zone have a profound impact on the subsequent landslide movement. Based on this, loess-mudstone mixtures with varying mudstone content were created, and by doing so, a more comprehensive understanding of the change in soil water retention capacity along the longitudinal section direction was gained.

**Figure 6:**
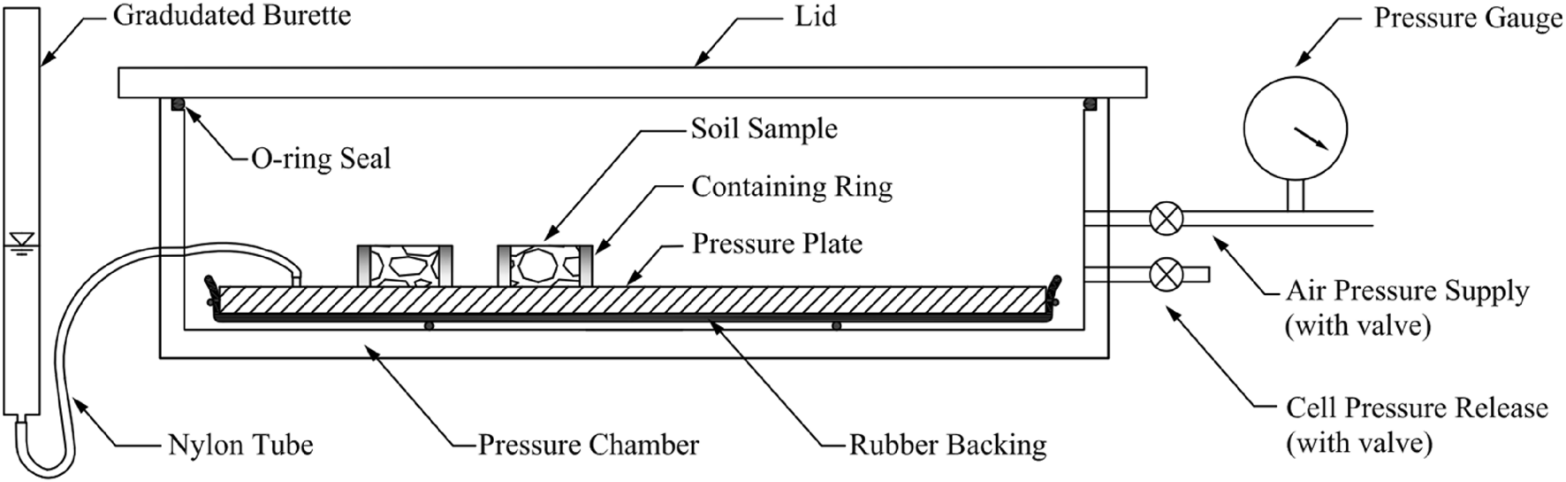
Schematics of the pressure plate apparatus.

The soil water characteristics curves of prepared samples are obtained via pressure plate method. The pressure plate method employs a porous ceramic plate with a high air entry value (AEV) to uphold a pressure gradient between the pore water pressure (${u}_{{\text{w}}}$) within the plate and the externally applied air pressure (${u}_{{\text{a}}}$). During the experiment, saturated soil samples are positioned on the porous plate within a sealed chamber, initiating the drainage of pore fluid from the soil samples through the plate, propelled by the established pressure difference. Once equilibrium is reached, the soil samples resting on the plate exhibit a matric suction that corresponds to the pressure disparity between the applied ${u}_{{\text{a}}}$ and ${u}_{{\text{w}}}$. Subsequently, the water content of the samples can be determined. By iteratively conducting this procedure with various air pressures, a plot representing the soil water characteristic curve can be derived. The establishment of equilibrium for each pressure level necessitates a minimum duration of one week, so it should be noted that to obtain a complete SWCC, encompassing an air pressure increment from 0 to 1500 kPa, a timeframe of approximately two months is typically required. Although the pressure plate method is the lengthy and time-consuming, it is advantageous over other methods as it allows for a direct and reliable control over the matric suction within the sample. A typical schematic of pressure plate equipment is presented in Fig. 6.

Prior to conducting the tests, the pressure plate underwent a saturation process. This involved immersing it in de-aired and distilled water for more than 24 h, followed by subjecting it to a vacuum of −100 kPa for a minimum of 4 h. Once saturated, the plate was carefully positioned in the chamber, ensuring its surface was covered with a thin layer of water to maintain saturation. To initiate the test, saturated soil samples were placed on the porous ceramic plate within the chamber. The chamber was then closed, and the first target air pressure was applied. As the applied air pressure increased, it forced the pore water inside the sample to pass through the plate and collect in the burette. This process continued until the matric suction within the sample equaled the applied air pressure. Throughout the test, the water level in the burette was regularly monitored to track progress towards equilibrium. Adjustments were made to the vertical position of the burette to maintain the same level as the soil samples. Each air pressure level was maintained for at least 7 days to ensure equilibrium was reached. After equilibrium was attained, the valve connected to the burette was closed to prevent water from returning to the ceramic plate. Subsequently, the air pressure was reduced to zero, and the chamber was opened to extract one of the samples for water content determination. Immediately after removal, the chamber was resealed, and the process was repeated with the application of the next air pressure level, following the same steps as previously described.

After the pressure plate data are obtained, they are then simulated and analyzed. Numerous formulas have been proposed to mathematically represent the soil water characteristic curve in the field of geotechnical and geological engineering. Among the most popular models are the Brooks and Corey model^30^, van Genuchten model^31^, and Fredlund and Xing model^32^, abbreviated as the BC model, VG model, and FX model, respectively, for simplicity. The BC model, being one of the earliest and simplest equations for SWCC, is only applicable to coarse-grained soils within relatively low suction ranges. On the other hand, the VG model incorporates a strong physical and theoretical basis, considering the pore size distribution of soil, and is suitable for a wide range of soil types across the entire suction range. The FX model, similar to the VG model, was developed by modifying the pore size distribution function provided by van Genuchten^31^. Considering its validity and simplicity, this study adopts the van Genuchten model to simulate the experimental data, which is expressed as follows.

1 $$\theta = {\theta }_{r}+\frac{({\theta }_{s}-{\theta }_{r})}{{\left[1+{(a\cdot s)}^{n}\right]}^{m}}$$

where ${\theta }_{r}$ and ${\theta }_{s}$ are residual and saturated volumetric water content, respectively, which can be accessed from SWCC data. ${\theta }_{r}$ corresponds to the minimum quantum of water retention within the soil matrix, while ${\theta }_{s}$ defines the upper threshold of the soil's capacity to retain water. a, n and m are model parameters, which are related to the inverse of air entry value, pore size distribution of soil and the overall symmetry of the soil water characteristic curve, respectively. These parameters can be determined by fitting the SWCC experimental data.

Hydraulic conductivity is an essential parameter for soil and water management related to agriculture^33,34^, the process assessment of infiltration, irrigation and drainage^35^, the establishment of hydrological models^36^, and for the geological disaster analysis such as slope instability, land subsidence, and ground fissures^37–39^. However, direct determination techniques in general tend to be labor-intensive, time-consuming and cost-inefficient^40^. In the case of unsaturated soil, the hydraulic conductivity is not a constant but rather varies depending on the water content and pore size distribution, which adds further complexity to the situation. Fortunately, this variability can be accurately described by statistical models based on SWCC. In the field of geotechnical engineering, in view of the good performance of the VG model in determining SWCC, the expressions of the hydraulic conductivity based on it is also widely accepted. The equation based on van Genuchten–Mualem function^31,41^ is given as

2 $${k}_{r}={k}_{s}{S}_{e}^{l}{\left(1-{\left(1-{{(S}_{e})}^\frac{1}{m}\right)}^{m}\right)}^{2}$$

3 $${S}_{e}=\frac{\theta -{\theta }_{r}}{{\theta }_{s}-{\theta }_{r}}$$

where ${k}_{r}$ is the unsaturated hydraulic conductivity, ${k}_{s}$ is the saturated hydraulic conductivity, ${S}_{e}$ is the effective saturation, and $l$ is a model parameter which can be determined by regression of the SWCC and a value of 0.5 is frequently taken.

To predict the unsaturated hydraulic conductivity, the saturated hydraulic conductivity needs to be determined in advance, which can be achieved experimentally according to Darcy's law.

4 $$v={k}_{s}i$$

where $v$ is the seepage velocity, ${k}_{s}$ is the saturated hydraulic conductivity, and $i$ is the hydraulic gradient, defined as the ratio of the head difference between the two points to the seepage length between the two points, i.e. $i=H/L$, when seepage occurs between any two points in the soil.

**Figure 7:**
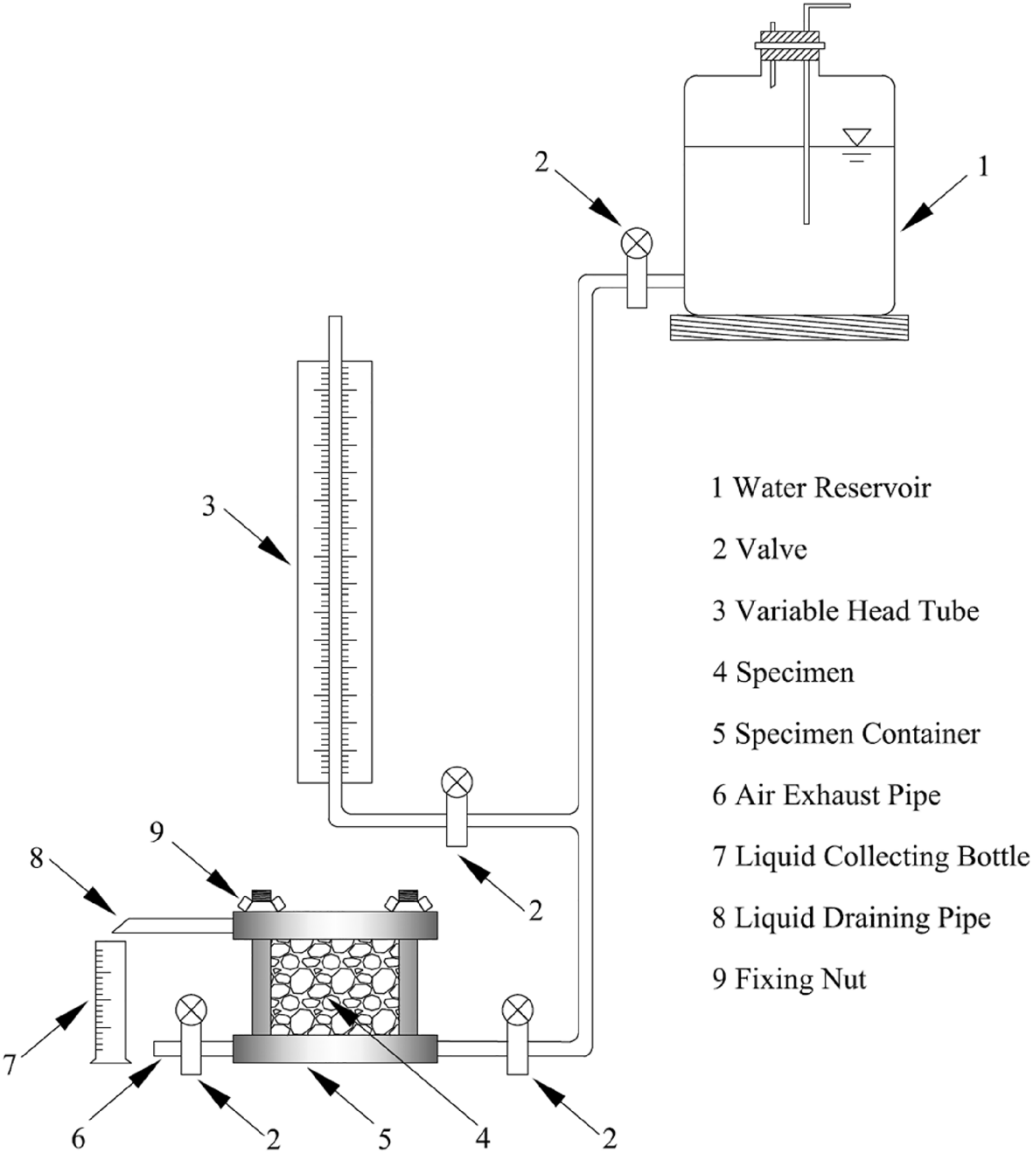
Schematic of the setup of variable head penetration test.

To do so, the variable head penetration test was conducted on the undisturbed loess samples extracted from the depths of 3 m, 5 m, and 7 m by using TST-55 permeameter^42–44^. A schematic of the test setup is presented in Fig. 7. The essential principle of this test is that the quantity of water descending within the variable head pipe should equate to the amount of water passing through the soil sample when the sample is completely saturated. The changing amount of water in the variable head pipe can be obtained by recording the water head at the beginning and end of the test. Assuming that after dt time, the water level in the variable head pipe decreases by dh, then the amount of water flowing through the soil sample during dt time can be expressed as

5 $$d{q}_{1}=-adh$$

where the negative sign indicates that the water volume increases as dh decreases. According to Darcy’s Law, the amount of water seeping out of the sample within dt time is

6 $$d{q}_{2}=kiAdt=k\frac{\Delta H}{L}Adt$$

Given that $d{q}_{1}=d{q}_{2}$, saturated hydraulic conductivity can be calculated by combining the Eqs. (5) and (6), and conducting integration, which is determined as

7 $${k}_{s}=2.3\frac{aL}{A({t}_{2}-{t}_{1})}lg\frac{{H}_{1}}{{H}_{2}}$$

where $a$ is the cross-sectional area of the variable water head pipe, $L$ is the penetration path, that is, the height of the sample, $A$ is the cross-sectional area of the sample, ${t}_{1}$, ${t}_{2}$ are the starting and ending times of measuring the water head, and ${H}_{1}$, ${H}_{2}$ are the starting and ending water heads.

## Test results and discussion

### SWCC test

**Figure 8:**
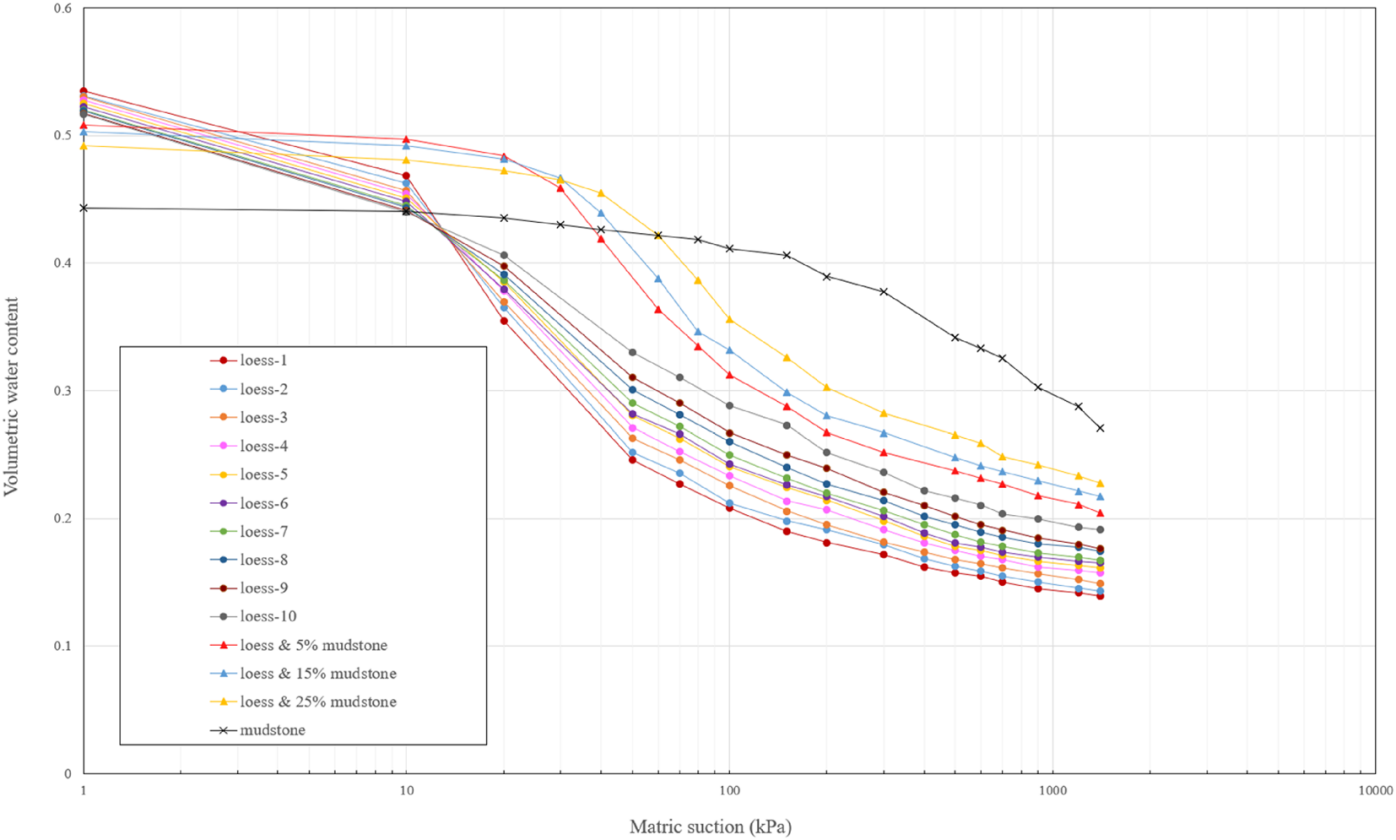
SWCC data for samples of loess, mudstone and loess-mudstone mixture.

The experimental data obtained by the pressure plate test is shown in Fig. 8. Given the extensive number of results and the clustered distribution of data points, the experimental data within each group are connected by polylines, which allows for convenient observation of the changing trend of each group. According to the experimental findings, it can be observed that the SWCC distribution patterns of the loess samples obtained at different depths are essentially similar. The variations among the curves mainly occur in the unsaturated segment, where a slight upward shift is observed as the depth increases. This upward shift in the curves is attributed to the changes in soil compaction or porosity ratio with increasing depth^45–49^. Regarding the phenomenon of the inconspicuous differences between the curves, it primarily stems from the stress release occurring in the soil on the back wall of the landslide. Furthermore, the occurrence of tensile cracks during stress release creates favorable channels for weathering, allowing elements like surface water, airflow, and biological effects to directly penetrate the soil and induce weathering. Consequently, the outcome of this process is the diminished significance of variations in soil density with depth^4,7,9^. It is worth noting that all tested loess samples exhibit similar air entry values, falling within the range of 10–20 kPa. The air entry value signifies the crucial suction point that distinguishes between saturated and unsaturated states. A lower air entry value implies that the soil can more readily attain full saturation. This characteristic poses significant challenges for the loess near the contact surface with mudstone, particularly during the rainy season or when there is increased irrigation water. During such periods, surface water rapidly infiltrates through the tensile cracks and accumulates at the mudstone interface, leading to complete saturation of the loess. This scenario raises concerns of potential landslides at the loess-mudstone contact surface.

**Figure 9:**
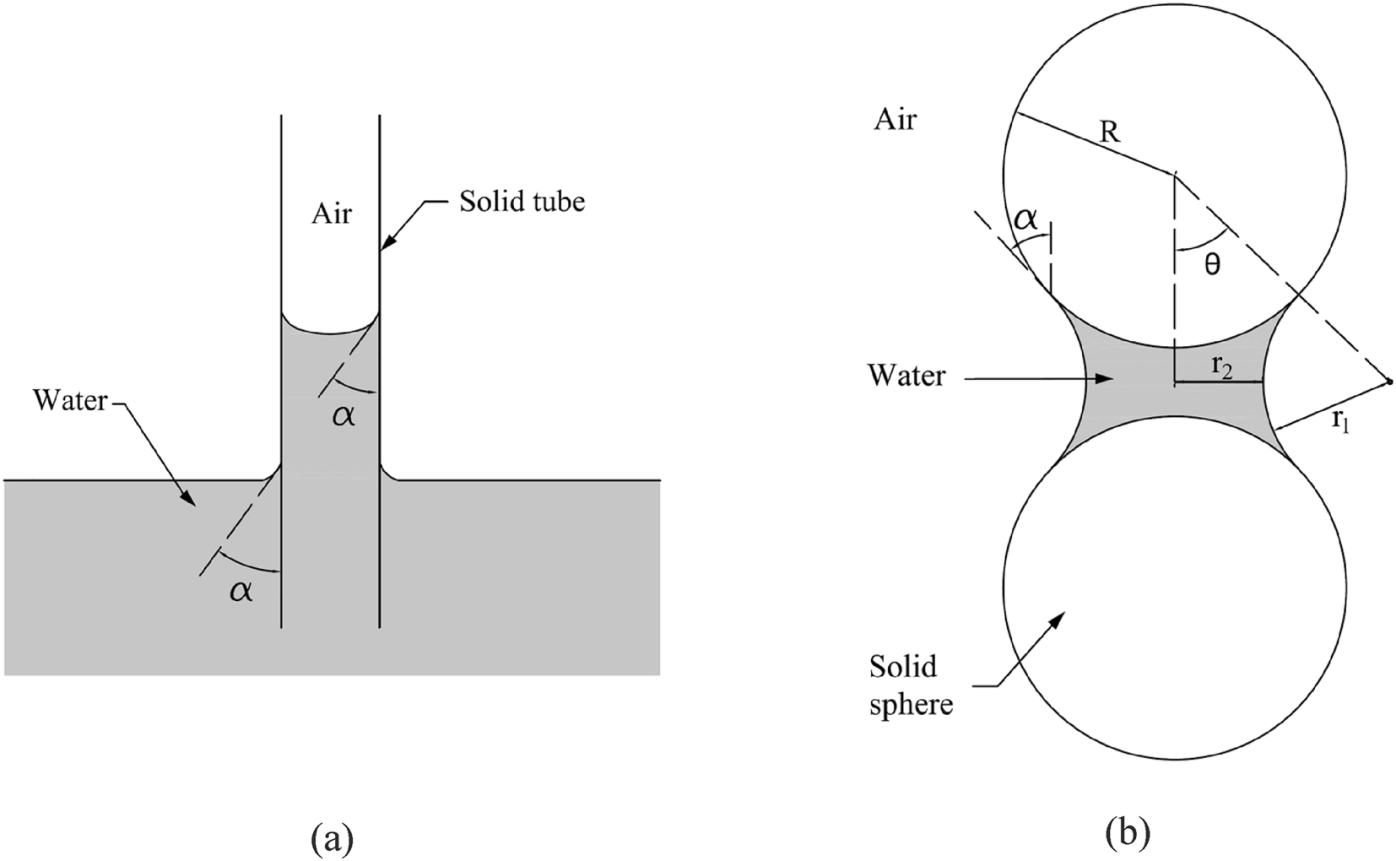
Schematic of capillary action (**a**) capillary tube, (**b**) contacting spheres.

The SWCC of mudstone shows a notable contrast with loess, characterized by a more pronounced water holding capacity and a higher air entry value, approximately 220 kPa. These findings align with the higher clay content observed in the mudstone during the particle size distribution analysis. Capillary action in the soil is one of the primary mechanisms responsible for the formation of soil water retention capacity, and this action is intricately linked to the diameter of the voids or soil particles^50^. Taking a simple capillary tube model as an example, as shown in Fig. 9a, the relationship between tube radius and matric suction can be expressed as

8 $$s={u}_{a}-{u}_{w}=\frac{2{T}_{s}}{r/cos\alpha }$$

where *s* is matric suction, ${u}_{{\text{a}}}$, ${u}_{w}$ are air pressure and pore water pressure respectively, ${T}_{s}$ is the surface tension, $r$ radius of capillary tube, and α is the contact angle between water and soil particle.

As for contacting sphere model with identical diameters, as shown in Fig. 9b, the mathematical expression describing the relationship between particle size and matric suction is given as

9 $$s={u}_{a}-{u}_{w}={T}_{S}\left(\frac{1}{{r}_{1}}-\frac{1}{{r}_{2}}\right)$$

where *s*, ${u}_{a}$, and ${u}_{w}$ have the same meaning in Eq. (2). ${r}_{1}$ and ${r}_{2}$ are parameters describing the meniscus between the two spheres, and they are functions of the radius of the sphere, *R*, as below

10 $${r}_{1}=R\left(\frac{1}{cos\theta }-1\right)$$

11 $${r}_{2}=R tan\theta -{r}_{1}$$

For other more complicated models, this similar inverse relationship between s and r can be found. Concisely stated, under the same conditions, a decrease in soil particle size correlates with an increase in matric suction and a heightened water retention capacity.

In addition, it is worth noting that the volumetric moisture content of the mudstone sample undergoes a significant reduction when saturated. This reduction can be mainly attributed to the mudstone sample's higher dry density and smaller void ratio, resulting in a smaller pore volume compared to the loose loess sample. Consequently, the total water storage capacity of the mudstone sample is correspondingly less than that of the loess sample. However, it is important to observe that despite these differences, the water retention capacity of mudstone quickly surpasses that of loess when examining the Fig. 7. When the matric suction in the soil is greater than 20 kPa, a suction value that is often easily reached, the water holding capacity of mudstone outperforms that of loess. This implies that mudstone can retain a higher volumetric moisture content under the same suction conditions. The distinct soil water characteristic curves exhibited by loess and mudstone further support the need to investigate the combination of these two materials.

It is evident from the experimental results that the SWCC distribution of the mixed sample exhibits a transitional pattern between mudstone and loess SWCC. Moreover, as the mudstone content increases in the mixture, the water retention capacity gradually increases as well. This phenomenon is very detrimental to loess-mudstone landslides, as loess with a higher mudstone content can retain more water under the same matric suction conditions. Consequently, when the sliding zone soil at the contact surface becomes a mixture of loess and mudstone due to sliding friction or other factors, the water retention capacity of the resulting mixture will be enhanced. In other words, more water can accumulate in this area, thereby creating potential hazards for the development and recurrence of landslides. As the process continues to unfold, and the mudstone content in the mixture increases further, the risk of landslide instability becomes even greater.

**Figure 10:**
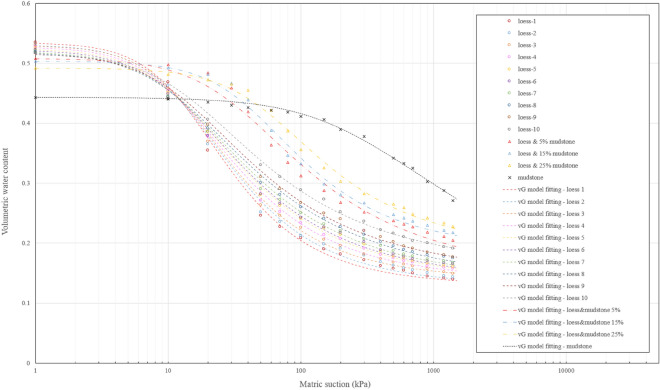
SWCC fitting curves by using VG model for samples of loess, mudstone and loess-mudstone mixtures.

In order to determine the mathematical expression of SWCC for each soil sample and enable continuous prediction of changes in water retention capacity, the van Genuchten model was employed to fit the experimental data. The fitting results are presented in the Fig. 10. It's evident that the VG model adeptly fits the SWCC data of diverse soil samples, capturing the variations in water retention capacity with minimal deviation. The corresponding parameters used for the fitting process are provided in the Table 2. The performance of the model is assessed by R^2^ and RMSE, as provided in the table. R^2^ is a statistical measure that represents the proportion of the variance in the dependent variable that is predictable from the independent variable in a regression model. It ranges from 0 to 1, with higher values indicating a better fit of the model to the data. Root Mean Squared Error (RMSE) is a commonly used metric to measure the differences between predicted values and the actual observed values, and lower RMSE indicates a better fit of the model to the data.

**Table 2:** Fitting parameters used by VG model for samples of loess, mudstone and loess-mudstone mixtures.

Sample	Model parameters			R^2^	RMSE
	a	n	m		
Loess 1	0.0724	1.8494	0.4593	0.9711	0.0224
Loess 2	0.0733	1.7784	0.4377	0.968	0.0232
Loess 3	0.0727	1.7862	0.4402	0.9754	0.0198
Loess 4	0.0756	1.7044	0.4133	0.9753	0.0192
Loess 5	0.0759	1.6517	0.3946	0.9766	0.0183
Loess 6	0.0768	1.6274	0.3855	0.9779	0.0178
Loess 7	0.0766	1.5924	0.3720	0.9796	0.0162
Loess 8	0.0788	1.5413	0.3512	0.9815	0.0153
Loess 9	0.073	1.5586	0.3584	0.9842	0.0139
Loess 10	0.068	1.529	0.3460	0.9871	0.0121
Loess & 5% mudstone	0.0316	1.543	0.3519	0.9841	0.0143
Loess & 15% mudstone	0.0239	1.7325	0.4228	0.9924	0.0092
Loess & 25% mudstone	0.0181	1.6645	0.3992	0.9943	0.0077
Mudstone	0.0046	1.2407	0.1940	0.9945	0.0044

**Figure 11:**
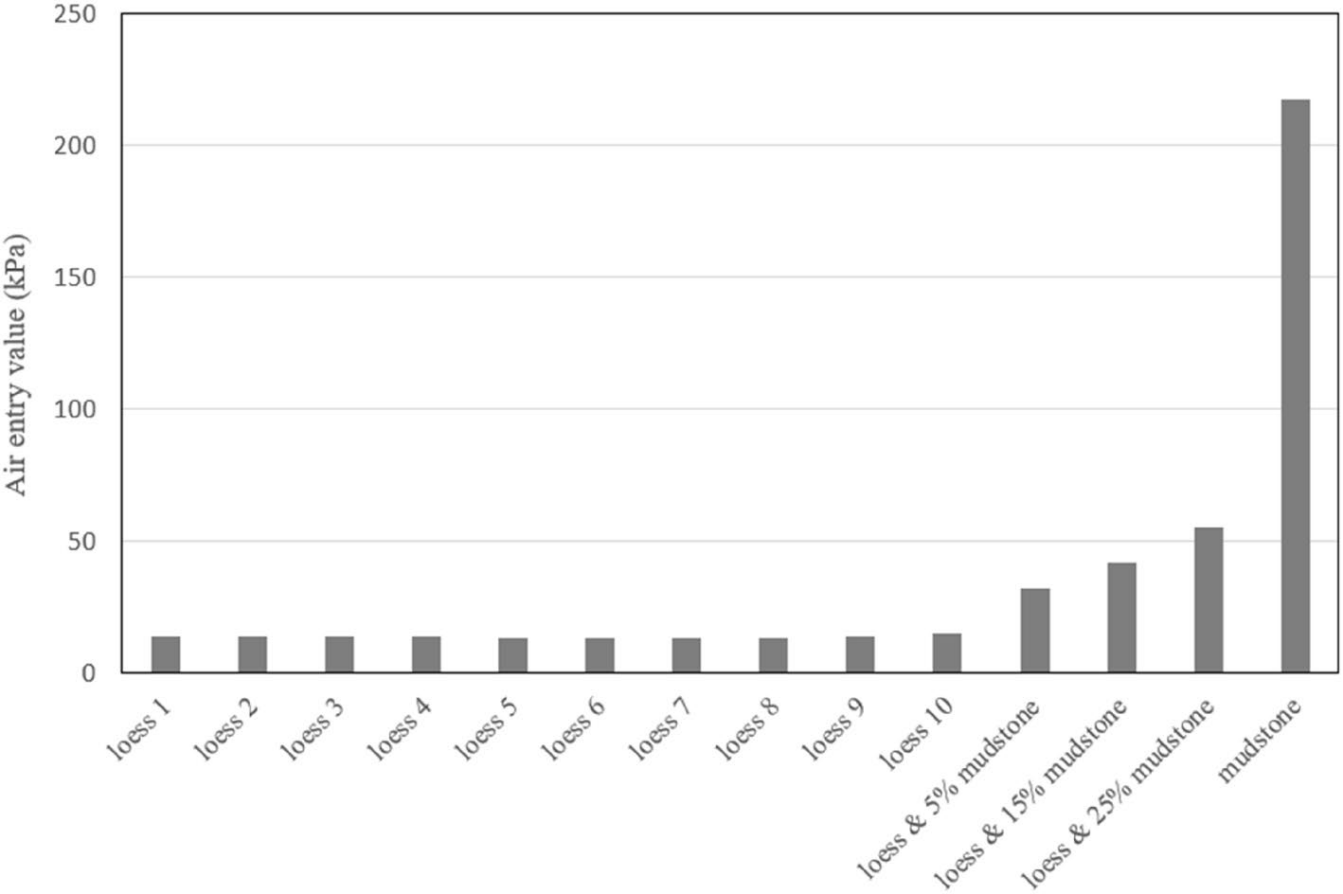
Air entry values for samples of loess, mudstone and loess-mudstone mixtures.

The parameter 'a' in the VG model represents the inverse of the air entry value, which allows to infer the air entry value of the soil sample from the fitting results. The air entry values obtained from experimental data and fitting results are shown in Fig. 11. It is evident that the air entry values of loess soil samples at different depths remain relatively consistent. Furthermore, as mudstone is mixed in, the air entry value gradually increases. Compared to loess, pure mudstone exhibits significantly higher air entry values. These findings align with expectations and are congruent with the experimental results and real scenario.

Accurate determination of SWCC not only provides an intuitive understanding of soil water retention capacity, but also contributes to other important aspects related to unsaturated soil. In the natural environment, the soil above the groundwater level is mostly unsaturated, especially in conditions where water infiltration is limited. Understanding the physical and mechanical properties of the soil during this phase holds significant value in comprehending landslides' development and their potential impact on overall stability. Thus, studying the SWCC has theoretical and practical significance for these purposes. Numerous experimental and theoretical studies have consistently affirmed the crucial role of the soil water characteristic curve (SWCC) in accurately modeling the behavior of unsaturated soils, such as determining permeability coefficient and effective stress^32,48,49,51–54^.

### Permeation test

The saturated hydraulic conductivity test results are presented in Table 3. The experimental results indicate that the saturated permeability coefficient of loess slightly decreases with increasing depth. However, it remains within the same order of magnitude, suggesting that the permeability of loess at different depths is comparable at this particular time. Furthermore, the values are greater than the typical permeability coefficient values reported for intact Malan loess in the literature, which is around 0.2 mm/min^55^. This observation can be primarily attributed to stress release resulting from landslides and subsequent erosion^4,56^, leading to a looser structure of the loess and reducing differences along the depth. This finding aligns well with other experimental results obtained in this study.

**Table 3:** Saturated hydraulic conductivity for loess sample at different depths.

Samples	Loess 3	Loess 5	Loess 7	Average
Saturated hydraulic conductivity, ${k}_{s}$ (mm/min)	1.06	1.02	1.01	1.03

**Figure 12:**
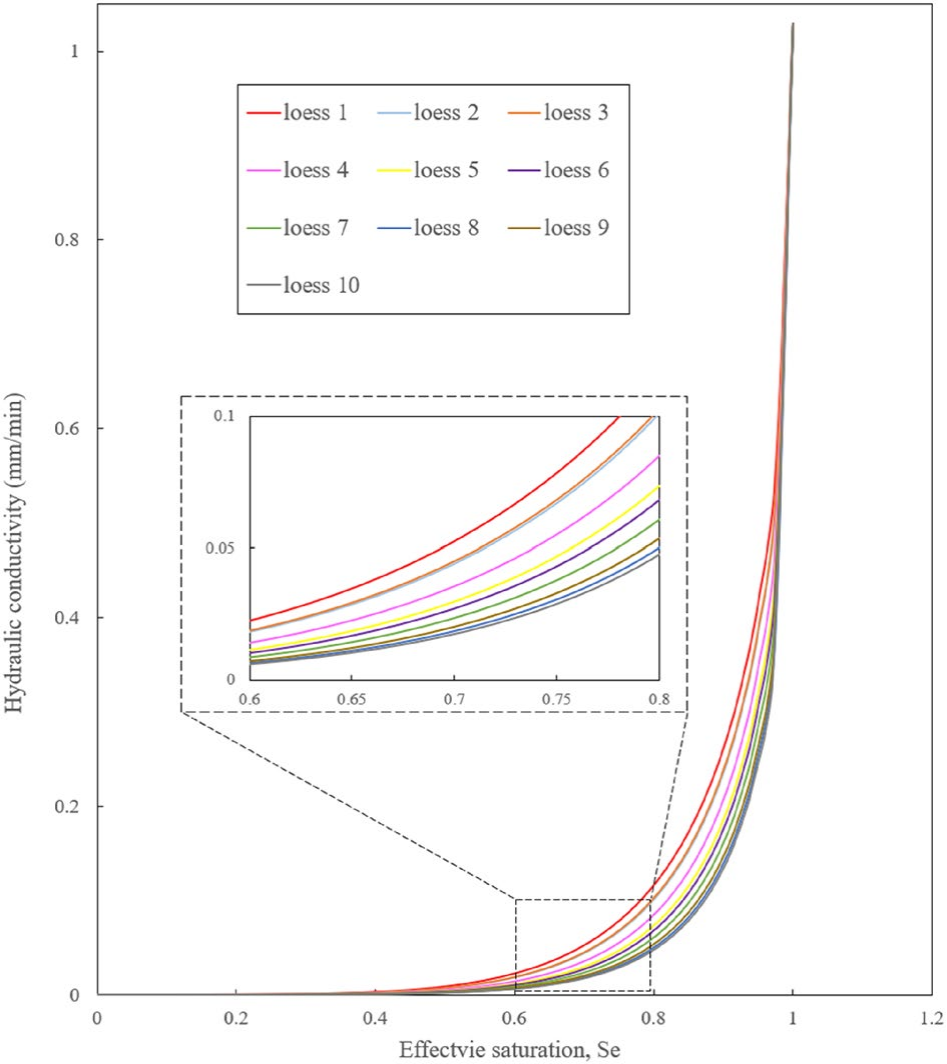
Predicted hydraulic conductivity for unsaturated loess samples.

Given the minimal variation in the saturated permeability coefficient with depth, it is rational to take the average value as the permeability coefficient of loess at different depths on the profile. By combining this information with the SWCC as well as relevant fitting parameters derived from this study, it becomes feasible to estimate the unsaturated permeability coefficient of the soil, as presented in Fig. 12.

The concept of effective stress serves as a fundamental principle in soil mechanics, exerting a significant influence on the assessment of shear strength in both soils and rocks, as well as the evaluation of deformation caused by loading. This concept plays a crucial role in various disciplines including slope stability analysis, foundation design, and estimation of settlement. The most widely accepted expression of effective stress extended for unsaturated soil takes the form proposed by Bishop (1959) and Bishop and Blight (1963)

12 $${\upsigma }^{{{\prime}}}=\left(\upsigma -{{\text{u}}}_{{\text{a}}}\right)+\upchi ({{\text{u}}}_{{\text{a}}}-{{\text{u}}}_{{\text{w}}})$$

in which ${\upsigma }^{{{\prime}}}$ is the effective stress, ${{\text{u}}}_{{\text{a}}}$ and ${{\text{u}}}_{{\text{w}}}$ are pore air pressure and pore water pressure, respectively. $\upchi$ is the effective stress parameter, which describes the attribution of matric suction to effective stress, and it takes the value of 1 for saturated soils and 0 for completely dry soils. The accurate determination of the effective stress parameter, $\upchi$, plays a pivotal role in establishing the effective stress concept. To address this, extensive research was conducted by Khalili and Khabbaz in 1998, wherein they systematically analyzed a vast array of shearing test data obtained from various soils documented in the literature. Their study culminated in the formulation of an equation relating the value of $\upchi$ to the air entry value of soil, which can be derived from the SWCC. The equation is given as

13 $$\chi =\left\{ \begin{array}{c} 1\quad for\quad \frac{s}{{s}_{e}}\le 1\\ {\left(\frac{s}{{s}_{e}}\right)}^{-0.55}\quad for\quad 1 \le \frac{s}{{s}_{e}} \le 12\end{array}\right.$$

where ${\text{s}}$ is the actual matric suction existing in soil, ${{\text{s}}}_{{\text{e}}}$ is the suction value separating the saturated and unsaturated states, which are often denoted as air entry value or air expulsion value according to hydraulic path the soil traces. By leveraging the SWCC acquired via this study and the corresponding air entry value, it becomes feasible to derive both the matric suction and the $\upchi$ value for any hydraulic state, that is, any moisture content levels. Consequently, this comprehensive approach facilitates the determination of the effective stress of the unsaturated soil.

### Microstructural observation

**Figure 13:**
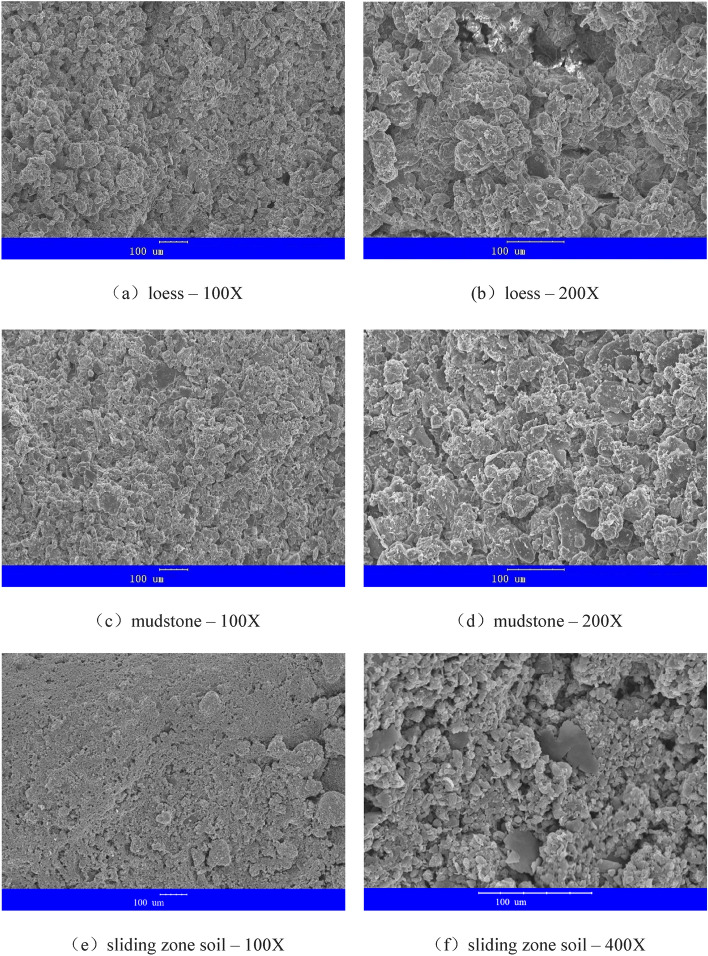
SEM test results for in-situ loess, mudstone and sliding zone soil.

Various properties of soil are closely related to its structure. To gain a comprehensive and profound understanding of loess-mudstone landslides, SEM observations were conducted on soil samples extracted from in-situ loess, mudstone, and the sliding zone, and the experimental findings are depicted in the Fig. 13. The loess soil particles primarily exhibit a granular nature, with the presence of relatively large pores. On the other hand, the mudstone particles display a denser and more compact distribution. As mudstone contains more clay minerals such as chlorite, more flaky clay structures can be observed. Notably, the sliding zone soil, which is sandwiched between the two aforementioned types, shows significant differences in the SEM images. Figure 13e reveals a compelling visual representation indicating the overall disruption of the soil particle framework, manifesting a flattened state. Upon closer inspection at 400-fold magnification in Fig. 13f, the soil particles are identified as fractured. Intriguingly, the soil also exhibits the formation of paper-like flakes resulting from the crushing friction.

**Figure 14:**
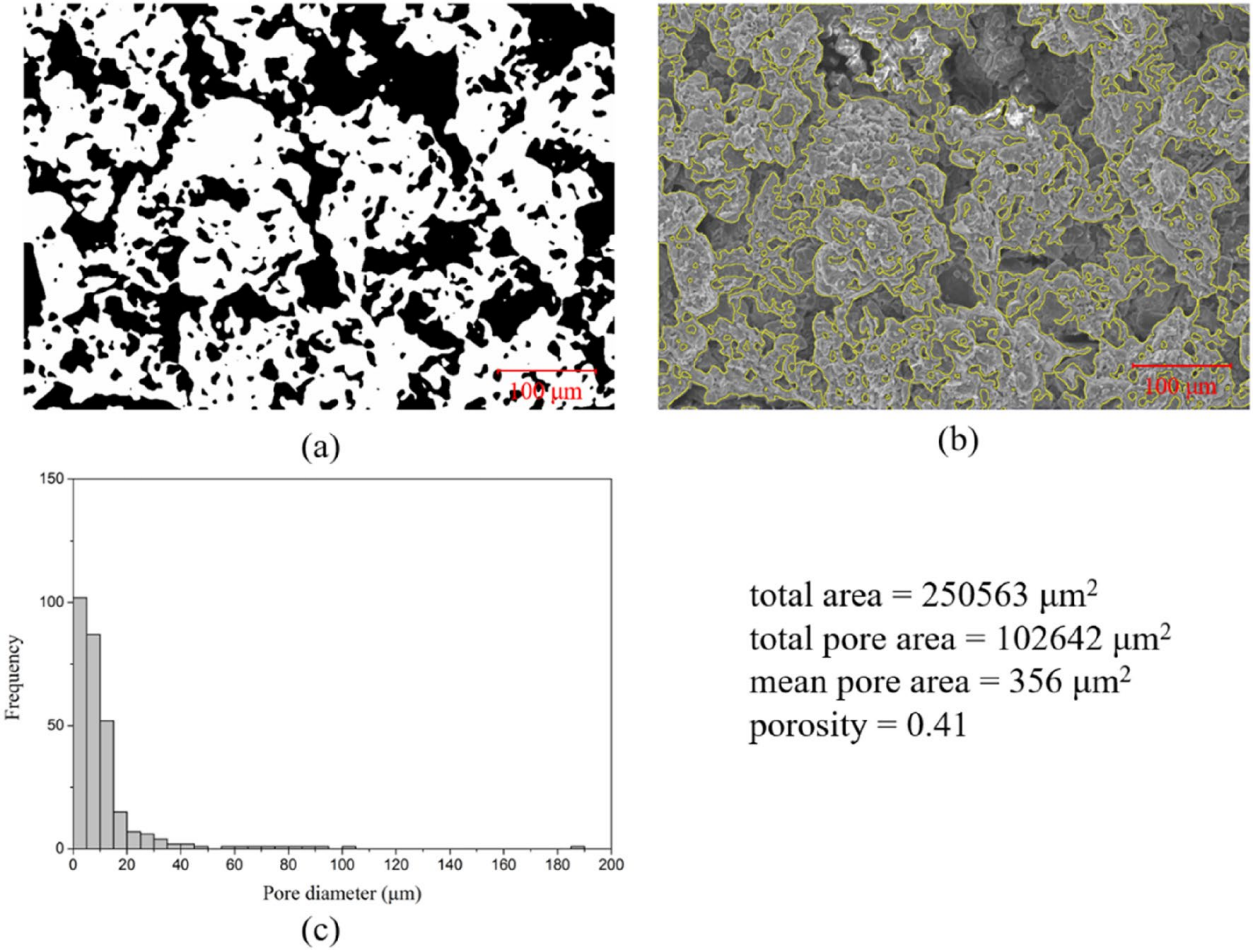
SEM image analysis of loess: (**a**) binarization image with pores in black and soil particles in white; (**b**) comparison of the processed boundary and the actual SEM image; (**c**) histograms of pore diameter distribution.

**Figure 15:**
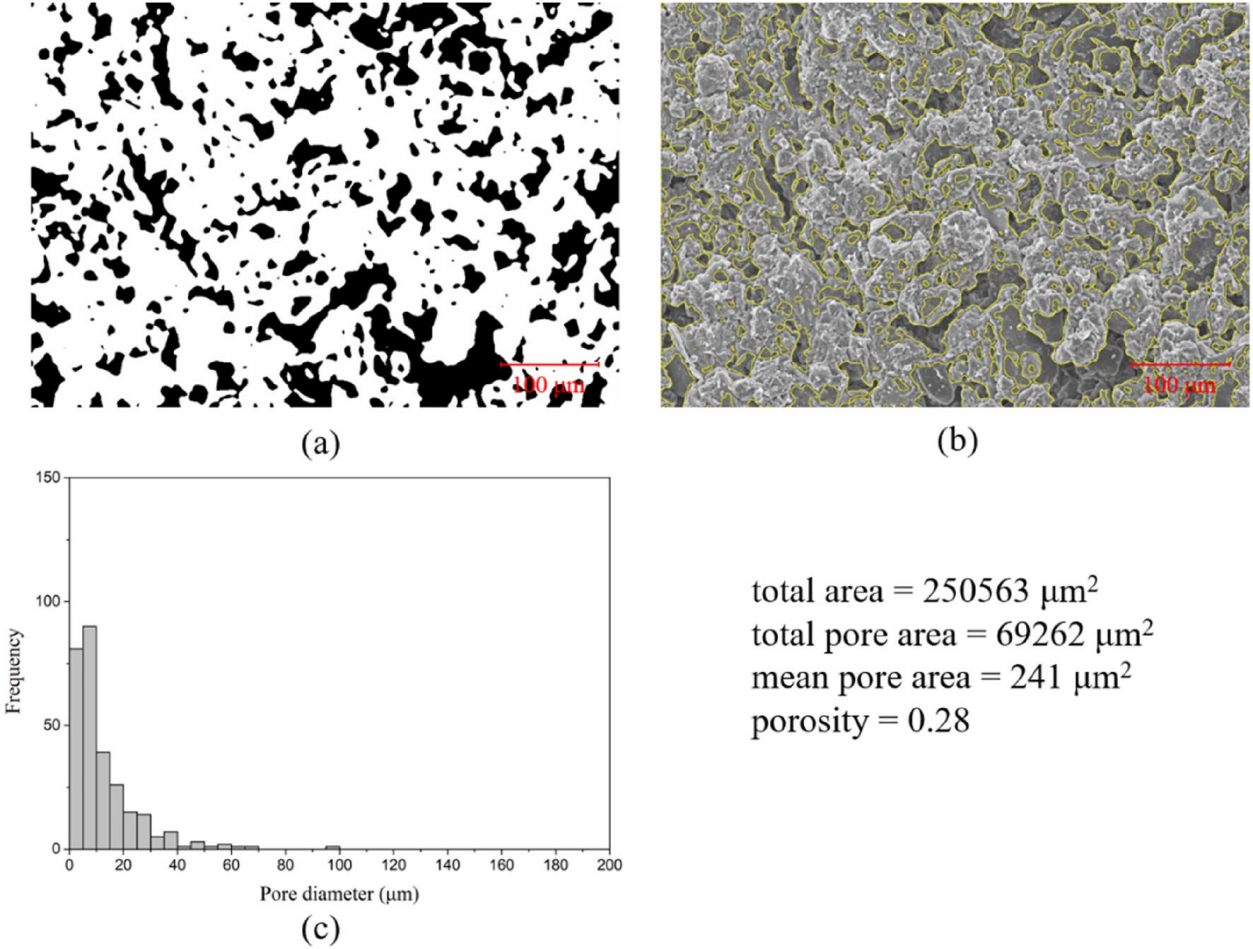
SEM image analysis of mudstone: (**a**) binarization image with pores in black and soil particles in white; (**b**) comparison of the processed boundary and the actual SEM image; (**c**) histograms of pore diameter distribution.

Graphic techniques such as binarization facilitate the observation and quantitative assessment of particles and voids within soil samples. Consequently, SEM images of loess and mudstone soil samples at a 200 × magnification, as shown in Fig. 13b and d, were subjected to such graphic processing, with the outcomes depicted in Figs. 14 and 15, respectively. In the resultant binarized images, Figs. 14a and 15a, the soil particles are presented in white, whereas the voids are rendered black. To enable an intuitive evaluation of the image processing's efficacy, the delineated particle boundaries were superimposed onto the original photographs for comparative purposes. Concurrently, a statistical analysis of the processed soil voids was undertaken, culminating in the construction of a histogram to supply data for quantitative analysis. The results affirm that the graphic processing proficiently captures the principal pores within the SEM images. A comparative evaluation reveals that loess contains a greater number of voids, which are, on average, larger than those in mudstone. Furthermore, loess exhibits some voids with considerably larger apertures. Collectively, these findings suggest that loess possesses a more porous and structurally looser composition in comparison to mudstone.

Based on the SEM images and corresponding quantitative analysis, it is evident that loess exhibits a higher quantity of voids, a larger average pore diameter, and greater porosity compared to mudstone. The presence of larger voids results in reduced matric suction in the soil under identical conditions, leading to decreased water holding capacity. The loose and porous structure make it conducive for water flow, consequently yielding a higher permeability coefficient. The microstructure observed by SEM is in good agreement with the experimental findings carried out in this study. The voids observed in loess and the resulting loose structure are consistent with the results obtained from the SWCC test, demonstrating the low water retention capacity of loess. The flaky structure developed in the mudstone echoes the higher clay mineral content identified through PSD test and XRD analysis. Furthermore, the uniform and dense distribution of the soil skeleton in mudstone is in agreement with the SWCC experimental outcome, indicating a higher water retention capacity. The SEM images of the slip zone soil vividly depict the intense movement occurring at the contact surface during a loess-mudstone landslide. These images further support the presence of friction and mixing between loess and mudstone throughout the sliding process, highlighting the importance of investigating the mixture of loess and mudstone designed in this study.

## Conclusion

The occurrence of landslides is dependent on various interrelated factors, among which the influence of water is particularly significant. This study focuses on the most common type of landslide in loess areas, namely loess-mudstone landslides, and investigates the alterations in hydraulic properties at different depths, which aims to advance the understanding of this specific landslide type and provide novel research direction. To do so, the active Huanancun loess-mudstone landslide was selected as the research object and an in-depth geological survey was carried out. Furthermore, a comprehensive investigation was carried out on the loess, mudstone, and loess-mudstone mixtures encompassing a range of experimental techniques. These included the SWCC test using the pressure plate method, X-ray Diffraction (XRD) analysis, Scanning Electron Microscopy (SEM) observation, particle size distribution (PSD) analysis, along with fundamental geotechnical tests for determining soil parameters. Subsequently, the Van Genuchten model was utilized to effectively fit the SWCC data and establish a precise mathematical expression. The experimental results obtained exhibit a satisfactory level of concurrence with the observed phenomena in geological surveys and provide a rational elucidation for them.

Based on the experimental findings, it can be concluded that the particle composition of loess remains consistent at various depths, and the mineral composition is basically the same. In contrast, when comparing loess with mudstone, it becomes apparent that mudstone contains a greater abundance of fine-grained components and a higher concentration of clay minerals.

The distribution patterns of SWCC in the loess samples obtained at different depths are essentially similar, and they display comparable air entry values, ranging from 10–20 kPa. The primary variations among the curves are primarily observed in the unsaturated segment, which shows a slight upward shift as the depth increases. The deep loess in question displays comparable water retention properties to those observed in shallow weathered soil. This implies that, upon receiving an influx of water at the interface, saturation and subsequent degradation of the loess occur relatively effortlessly, leading to the initiation of landslides. As opposed to loess, mudstone exhibits commendable water retention capabilities and a high air entry value. The SWCC distribution of the mixture of these two materials displays a transitional trend between the SWCC of mudstone and that of loess. And as the mudstone content increases, the capacity of water retention augments accordingly. This also poses considerable harm to the stability of landslides, since in an unsaturated state, loess-mudstone mixture will hold more moisture than pure loess, which may serve as an underlying risk factor for the development and reoccurrence of landslides.

The fitting results of van Genuchten model indicate that it can accurately capture the variation in SWCC for different soil samples. The derived mathematical expression for SWCC and the associated AEV hold potential for utilization in various applications, such as estimating unsaturated permeability coefficients and calculating effective stress in soil. These findings contribute to the advancement of determining related parameters and establishment of constitutive models.

Furthermore, the employment of Scanning Electron Microscopy (SEM) provides insightful observations regarding the microstructural attributes of diverse soil samples. These include the identification of voids in loess, the observation of high clay content in mudstone, and the discernment of pronounced frictional characteristics in the sliding zone. Such SEM observations serve as a valuable complement to the experimental findings presented in this study.

In summary, this study reveals the variations in the hydraulic properties of soils in loess mudstone landslides along the depth and quantifies the characteristics of water retention and migration within them, which facilitates the development of preventive and remedial disaster management measures.

## Data Availability

The datasets used and/or analysed during the current study available from the corresponding author on reasonable request.
